# Simian Varicella Virus Infection and Reactivation in Rhesus Macaques Trigger Cytokine and Aβ40/42 Alterations in Serum and Cerebrospinal Fluid

**DOI:** 10.21203/rs.3.rs-3367215/v1

**Published:** 2023-10-16

**Authors:** Christy S Niemeyer, Vicki Traina-Dorge, Lara Doyle-Meyers, Arpita Das, Jayme Looper, Teresa Mescher, Brittany Feia, Eva Medina, Maria A. Nagel, Ravi Mahalingam, Andrew N. Bubak

**Affiliations:** University of Colorado Anschutz Medical Campus School of Medicine; Tulane National Primate Research Center; Tulane National Primate Research Center; Tulane National Primate Research Center; Louisiana State University School of Veterinary Medicine; University of Colorado School of Medicine: University of Colorado Anschutz Medical Campus School of Medicine; University of Colorado School of Medicine: University of Colorado Anschutz Medical Campus School of Medicine; University of Colorado School of Medicine: University of Colorado Anschutz Medical Campus School of Medicine; University of Colorado School of Medicine: University of Colorado Anschutz Medical Campus School of Medicine; University of Colorado School of Medicine: University of Colorado Anschutz Medical Campus School of Medicine; University of Colorado School of Medicine: University of Colorado Anschutz Medical Campus School of Medicine

**Keywords:** Simian Varicella Virus, Cytokine, Amyloid-beta, Varicella, Zoster

## Abstract

Simian varicella virus (SVV) produces peripheral inflammatory responses during varicella (primary infection) and zoster (reactivation) in rhesus macaques (RM). However, it is unclear if peripheral measures are accurate proxies for central nervous system (CNS) responses. Thus, we analyzed cytokine and Aβ42/Aβ40 changes in paired serum and cerebrospinal fluid (CSF) during the course of infection. During varicella and zoster, every RM had variable changes in serum and CSF cytokine and Aβ42/Aβ40 levels compared to pre-inoculation levels. Overall, peripheral infection appears to affect CNS cytokine and Aβ42/Aβ40 levels independent of serum responses, suggesting that peripheral disease may contribute to CNS disease.

## Introduction

Varicella-zoster virus (VZV) is an alphaherpesvirus that exclusively infects humans, with a global infection rate of nearly 90% (Gershon et al. 2015). Initial exposure to VZV leads to primary infection, characterized by a disseminated rash known as varicella (chickenpox), during which the virus infects and establishes latency in cranial and spinal ganglionic neurons (Mahalingam et al. 1990; Richter et al. 2009; Zerboni et al. 2014). As individuals age or experience immunosuppression, VZV can reactivate, resulting in the typical dermatomal-associated skin rash known as shingles (zoster). In rare cases, VZV can cause central nervous system (CNS) diseases such as encephalitis, meningitis, myelopathy, and vasculopathy (Gilden et al. 2010; Nagel et al. 2020). Given the rarity of these fulminant CNS manifestations, primary VZV infection and reactivation is largely considered a peripheral disease. Thus, the CNS response to primary infection and reactivation during the typical clinical VZV presentation is not well-characterized.

In this study, we used simian varicella virus (SVV) infection in rhesus macaques (RMs), a non-human primate model of human VZV infection, to describe the concurrent peripheral and CNS response during primary infection, latency, and reactivation (Mahalingam et al. 2022; Mahalingam et al. 2001; Mahalingam et al. 1990; Messaoudi et al. 2009). We measured cytokine and amyloid-β (Ab)-40 and − 42 levels in paired serum and cerebrospinal fluid (CSF) samples of six RMs inoculated with SVV. We confirmed previous reports of a proinflammatory peripheral response during primary infection and reactivation but also describe a dynamic CNS inflammatory response that, in some instances, preceded the peripheral response. These data suggest a transient yet robust CNS inflammatory response during all stages of VZV pathogenesis in the absence of an obvious fulminant lytic CNS infection.

## Methods

### Ethics Statement.

RMs used in this study were housed at the Tulane National Primate Research Center (TNPRC) in Covington, LA. All animal housing, care, and research were performed in compliance with The National Research Council, Guide for the Care and Use of Laboratory Animals and the Animal Welfare Act. The Tulane National Primate Research Center (TNPRC) is fully accredited by AAALAC International (Association for the Assessment and Accreditation of Laboratory Animal Care), Animal Welfare Assurance No. A4499-01. All studies were reviewed and approved by the Tulane University Institutional Animal Care and Use Committee (IACUC) under protocol number P0177. All clinical procedures were carried out under the direction of experienced laboratory animal veterinarians in the TNPRC Division of Veterinary Medicine. Clinical procedures were performed under anesthesia using approved anesthetics, and all efforts were made to minimize stress, improve housing conditions, and provide enrichment opportunities (e.g., objects to manipulate in cages, varied food supplements, foraging, and task-oriented feeding methods, and interaction with caregivers).

### SVV inoculation and establishment of latency.

Six SVV-seronegative adults, male, Indian RMs (Macaca mulatta), 7.0 years of age (KA59, KC22, LA16, LC23, LE26, LF30), were inoculated intratracheally with 1.5 × 10^5^ plaque-forming units (pfu) of wild type-SVV-infected rhesus lung fibroblast (Frhl-2) cells (Mahalingam et al. 1992). All RMs were anesthetized and monitored for well-being by physical exams with blood collections every 3 to 7 days and CSF samples, by cisterna magna punctures, collected weekly for the first two weeks and then biweekly thereafter until the establishment of latency. All six RMs developed a typical varicella rash within 7–10 days post-inoculation (dpi). Skin scrapings and punch biopsy (4 mm) samples from skin were collected upon the appearance of typical varicella rash lesions. Scrapings were processed for DNA (as shown below).

### Immunosuppressive treatment and euthanasia.

Four months after primary infection and clearance of SVV viremia, viral latency was confirmed in all RMs. At that time, four Experimental RMs (KA59, KC22, LA16, and LE26) were transported (from TNPRC in Covington, LA, USA, to the School of Veterinary Medicine, Radiation Oncology facility at Louisiana State University in Baton Rouge, LA). Once there, each RM was anesthetized and exposed to a single dose of 200-cGy total body X-irradiation and afterward treated daily with oral tacrolimus (Prograf; 80 µg/kg of body weight/day) and prednisone (2 mg/kg/day) to induce virus reactivation. All animals were monitored by physical exams for well-being and zoster rash with blood collections every 7 days post-treatment (dpX) and CSF collections every 7 or 14 dpX. Skin scrapings and punch biopsy (4 mm) samples were collected upon the appearance of typical zoster rash lesions and processed as described. The Treatment Control RM, LC23, was transported with the Experimental group but did not receive immunosuppressive treatment. Following SVV infection and latency, LF30, the SVV Latent Control RM, neither received immunosuppressive treatment nor was transported. All RMs were euthanized five months post-treatment.

### PBMC Isolation, DNA extraction, and qPCR.

EDTA anticoagulated blood samples were collected throughout acute infection, latency, treatment, and reactivation. Samples were centrifuged, plasma aliquoted, and peripheral blood mononuclear cells (PBMCs) isolated with Ficoll Hypaque^®^(Sigma-Aldrich) according to the manufacturer’s protocol for standard density gradient centrifugation. DNA was then isolated from the PBMCs using a commercial DNA extraction kit per the manufacturer’s instructions (Qiagen, Germantown, MD, USA), followed by quantitative PCR (qPCR) for SVV open reading frame (ORF) 61 DNA in PBMCs as described previously (Messaoudi et al., 2009).

### Proinflammatory and Aβ40 / Aβ42 ELISA.

RM Serum and CSF were analyzed in duplicate for pro-inflammatory cytokines (Interleukin [IL]-6, IL-2, IL-8, IL-10, IL-1β) using the non-human primate V-Plex pro-inflammatory panel per manufacturer’s instructions (Meso Scale Discovery, Rockville, MD) electrochemiluminescence immunoassay. Similarly, Aβ38 (data not shown), Aβ40, and Aβ42 were detected and quantified using the V-Plex Plus Aβ peptide panel one electrochemiluminescence immunoassay per manufacturer’s instructions (Meso Scale Discovery). Values of each analyte for each RM are displayed in supplemental Tables 1–6.

## Results

### Primary infection and reactivation profile of rhesus macaques.

**Figure 1:**
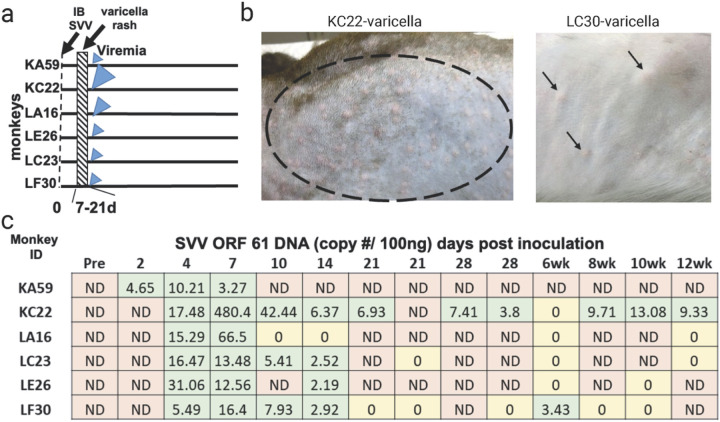
Timeline of SVV primary infection through reactivation. **a** six rhesus macaques (RM IDs: KA59, KC22, LA16, LE26, LC23, LF30) were intratracheally (IT) inoculated with SVV. These monkeys were separated into three groups; experimental (KA59, KC22, LA16, and LE26); transport control (LC23), which was transported to the facility but did not receive immunosuppression, and no transport-no immunosuppression control (LF30). All RMs developed varicella rash (indicated by dashed line) 7–21 days post-inoculation. RMs also developed viremia; blue arrows indicate the extent of viremia. **b** an example of varicella rash in KC22 (cranial dorsum and LF30 (upper back). **c** SVV ORF 61 DNA in the blood demonstrates the extent of viremia in all six RMs. Abv; ND= not detected, IT= intratracheal, SVV inoculation, d = days, wk = weeks, m = months.

**Figure 2:**
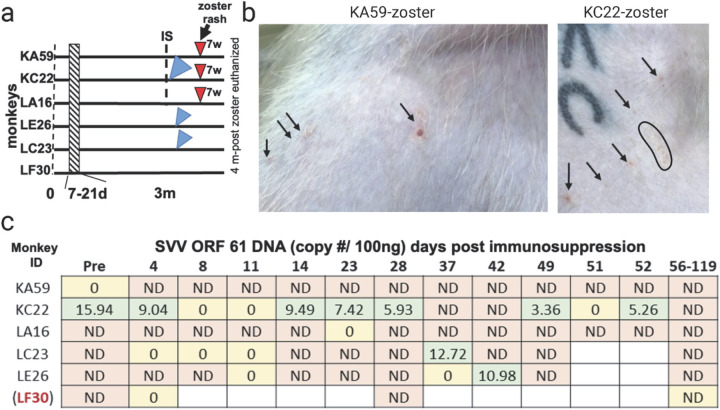
Timeline of SVV reactivation. **a** timeline of immunosuppression and reactivation of animals. Three animals (KA59, KC22, LA16) developed zoster (red arrows). All RMs were euthanized four months post-zoster. **b** an example image of zoster in KA59 (right shoulder) and KC22 (left shoulder). **c** SVV ORF 61 DNA was probed post-immunosuppression. Three RMs developed viremia: two experimental (KC22, LE26) and the transportation control (LC23). Zeros (yellow boxes) indicate only 1/3 triplicate runs detected SVV DNA; all other triplicates were averaged. Abv; ND= not detected, IS= immunosuppression, d = days, wk = weeks, m = months.

Six RMs were intrabronchially inoculated with SVV (Fig. 1a). All six animals developed varicella rash at 7–10 dpi (Fig. 1b), which was completely cleared by 21 dpi. Representative varicella rash in monkeys KC22 and LF30 are shown in Fig. 1b. Viremia was detected in one monkey (KA59) by 2 dpi and in the rest by 4 dpi. Viremia disappeared by 14 dpi in all monkeys except one (KC22), which persisted until 12 weeks post-inoculation. The establishment of latency was confirmed by the absence of SVV DNA in blood for two consecutive weeks (Fig. 1c). Latent infection was established in animals KA59, LA16, LC23, LE26, and LF30 by 21 dpi. Viremia persisted in animal KC22 for 12 weeks post-inoculation. (Fig. 1c). Three months post-inoculation, animals were immunosuppressed using one full-body X-irradiation and daily tacrolimus and prednisone treatment (Fig. 2a). Experimental animals (KA59, KC22, LA16, LE26) received X-irradiation and drug treatments. There were two control animals: LC23 was transported to the irradiation facility but neither received irradiation nor immunosuppressive drugs, and LF30 was not transported, nor immunosuppressed (Fig. 2a). Three experimental animals (KA59, KC22, LA16) developed zoster rash 7 weeks post immunosuppression. Representative zoster rash in monkeys KC22 and KA50 are shown in Fig. 2b. Two of the experimental animals (KC22, LE26) and the transportation control animal (LC23) had detectable SVV DNA in the blood at 49-, 42-, and 37-dpX, respectively (Fig. 2c).

### Changes in cytokine concentrations in serum and CSF during primary infection, latency, and reactivation.

#### IL-6 levels in serum and CSF.

**Figure 3:**
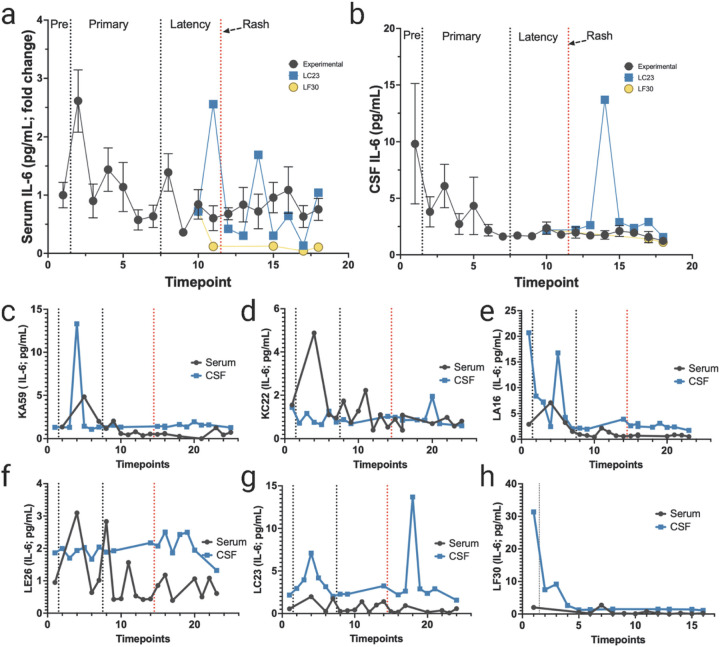
Changes in IL-6 in serum and CSF following primary SVV infection, latency, and reactivation. **a** fold change of IL-6 in serum (average for all RMs) from pre-SVV inoculation (Pre) for primary infection and latency. **b** average in CSF IL-6 (average for all RMs) from pre-SVV inoculation, primary infection, and latency. All RMs were treated the same during primary infection; only at transportation (timepoint 10) do LC23 and LF30 differ experimentally. **c-f**average fold change in serum (black) and CSF (blue) IL-6 can be seen for the four experimental RMs (KA59, KC22, LA16, LE26) following immunosuppression and reactivation (IV). **g, h** levels of IL-6 in serum LC23 (non-immunosuppressed) and LF30 (latently infected). Individual serum/CSF RM IDs are shown on the Y-axis. Individual serum or CSF IL-6 levels not detected are indicated as 0 in individual graphs.

Our initial goal was to determine differences in cytokines levels in the periphery versus the CNS. Serial blood draws, and spinal taps for CSF were obtained from each animal during pre-infection, primary infection, latency, and reactivation (Fig. 3). Similar to our previous studies, all animals that had blood drawn during viremia (LF30 data not available) showed a spike in serum IL-6 during primary infection (Fig. 3a; Traina-Dorge et al. 2014). Overall, IL-6 in the CSF showed a potential spike during primary infection. However, mean pre-inoculation levels were highly variable (Fig. 3b). Experimental animals KA59 (Fig. 3c), LA16 (Fig. 3e), and control monkey LC23 (Fig. 3g) showed spikes in IL-6 in the CSF during primary infection that tightly aligned with serum IL-6 levels. Experimental animals KC22 and LE26 showed large fluctuations in serum IL-6 levels during primary infection (Fig. 3d, f). Very little fluctuation was seen in the CSF of these two animals. However, there was a small spike in CSF IL-6 following zoster in KC22 (Fig. 3d). One non-immunosuppressed control animal, LC23, had detectable viremia during reactivation (Fig. 2c). It showed a large spike in CSF IL-6 without substantial change in serum IL-6 levels (Fig. 3g). There was no change in the IL-6 levels in serum or CSF during primary infection in the other latently infected control RM LF30 in serum (Fig. 3h).

#### IL-2 levels in serum and CSF.

**Figure 4:**
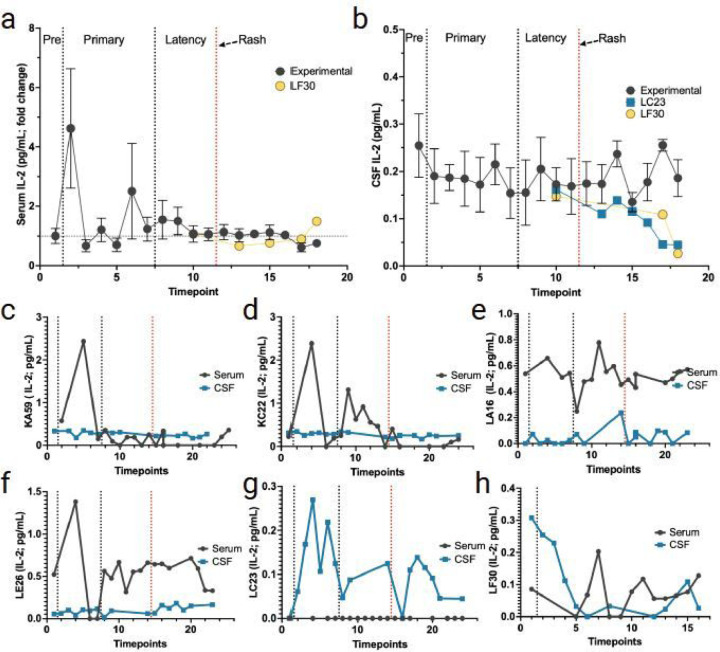
Changes in IL-2 in serum and CSF following primary SVV infection, latency, and reactivation. **a** fold change of IL-2 in serum (average for all RMs) from pre-SVV inoculation (Pre) for primary infection and latency. **b** average in CSF IL-2 means (for all RMs) from pre-SVV inoculation, primary infection, and latency. All RMs were treated the same during primary infection; only at transportation (timepoint 10) do LC23 and LF30 differ experimentally. **c-f** average fold change in serum (black) and CSF (blue) IL-2 can be seen for the four experimental RMs (KA59, KC22, LA16, LE26) following immunosuppression and reactivation. **g, h** levels of IL-2 in serum LC23 (non-immunosuppressed) and LF30 (latently infected). Individual serum/CSF RM IDs are shown on the Y-axis. Individual serum or CSF IL-2 levels not detected are indicated as 0 in individual graphs.

Similar to serum IL-6 levels during primary infection, three out of four experimental RMs (KA59, KC22, and LE26) had elevated spikes in the serum IL-2 levels (Fig. 4c, d, and f). However, very little change was seen in CSF IL-2 and remained largely unchanged in these experimental RMs (KA59, KC22, and LE26). Interestingly, IL-2 in LA16 did spike in the CSF before the onset of rash, with no change in serum (Fig. 4b–f). In addition, non-immunosuppressed RM (LC23) had a considerable spike in CSF IL-2 during primary infection, without any detectable levels of serum IL-2 (Fig. 4g). LC23 also showed an increase in IL-2 in CSF before the onset of viremia during varicella and post-zoster. The latently infected monkey LF30 also had a spike in serum IL-2, but (give the time) post varicella (Fig. 3h).

#### Serum and CSF levels of IL-10.

**Figure 5:**
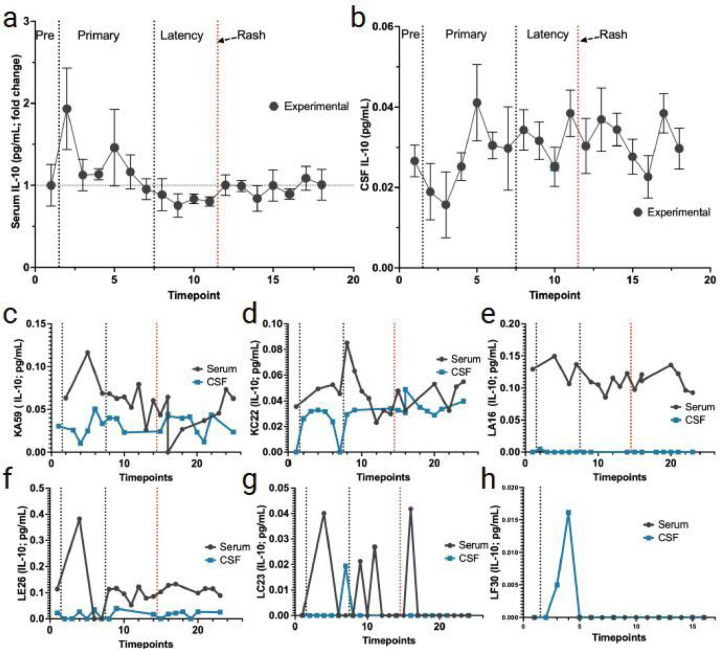
Changes in IL-10 in serum and CSF following primary SVV infection, latency, and reactivation. **a** fold change of IL-10 in serum (average for all RMs) from pre-SVV inoculation (Pre) for primary infection and latency. **b** average in CSF IL-10 (average for all RMs) from pre-SVV inoculation, primary infection, and latency. **c-f** IL-10 average fold change in serum (black) and CSF (blue) for the four experimental RMs (KA59, KC22, LA16, LE26) following immunosuppression and reactivation (IV). **g, h**levels of IL-10 in serum LC23 (non-immunosuppressed) and LF30 (latently infected). Individual serum/CSF RM IDs are shown on the Y-axis. Individual serum or CSF IL-10 levels not detected are indicated as 0 in individual graphs.

Overall IL-10 in experimental animals’ serum and CSF was elevated during primary infection with little overall change in latency and reactivation (Fig. 5a–b). Although lower levels of IL-10 were seen in CSF at early times during primary infection, they were more variable during latency and reactivation (Fig. 5b). Experimental animals (KA59, KC22, and LE26) and the non-immunosuppressed control monkey LC23 showed increased serum IL-10 during primary infection (Fig. 5c, d, f, g). Both control animals (LC23 and LF30) showed spikes of CSF IL-10 during primary infection (Fig. 5g, h). Further, LC23 also showed not only a spike in serum IL-10 levels during primary infection but also in latency and reactivation (Fig. 5g).

#### Serum and CSF levels of IL-1β.

**Figure 6:**
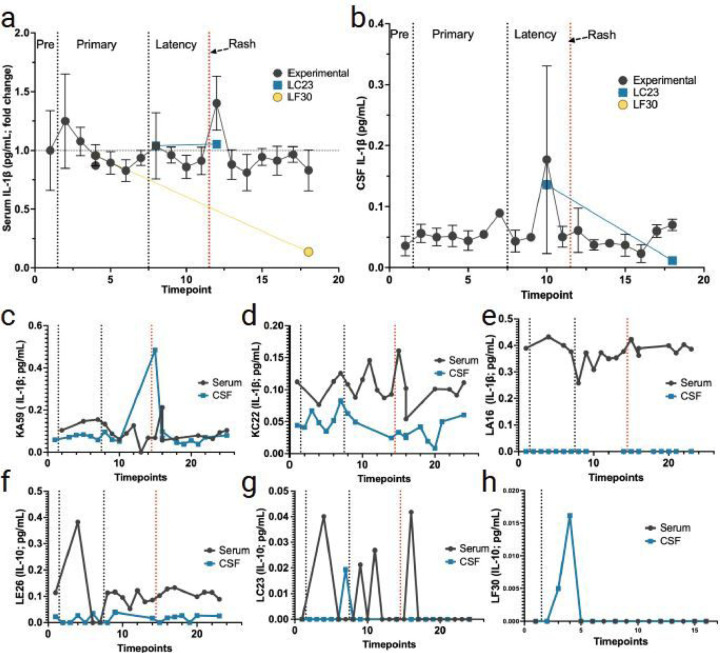
Changes in IL-1β in serum and CSF following primary SVV infection, latency, and reactivation. **a** fold change of IL-1β in serum (average for all RMs) from pre-SVV inoculation (Pre) for primary infection and latency. **b** average in CSF IL-1β (average for all RMs) from pre-SVV inoculation, primary infection, and latency. All animals were treated the same during primary infection; only at transportation (timepoint 10) do LC23 and LF30 differ experimentally. **c-f**average fold change in serum (black) and CSF (blue) IL-1β for the four experimental RMs (KA59, KC22, LA16, LE26) following immunosuppression and reactivation. **g, h** levels of IL-1β in serum LC23 (non-immunosuppressed) and LF30 (latently infected). Individual serum/CSF RM IDs are shown on the Y-axis. Individual serum or CSF IL-1β levels not detected are indicated as 0 in individual graphs.

Serum IL-1β only marginally increased during primary infection and then again during reactivation in the experimental animals (Fig. 6a). There was an increase in the average CSF IL-1β during latency preceding zoster onset in experimental animals (Fig. 6b). Experimental animals had higher variability in serum IL-1β (Fig. 6c–f). Of note, serum IL-1β level in experimental animal KA59 spiked preceding zoster, and this was followed by a larger spike in CSF IL-1β at the time of zoster (Fig. 6c). The non-immunosuppressed monkey (LC23) and the latently infected monkey LF30 had the most predominant spike in serum IL-1β following primary infection (Fig. 6g, h), while only LC23 had another spike in serum IL-1β following zoster (Fig. 6g).

#### Serum and CSF levels of IL-8.

**Figure 7:**
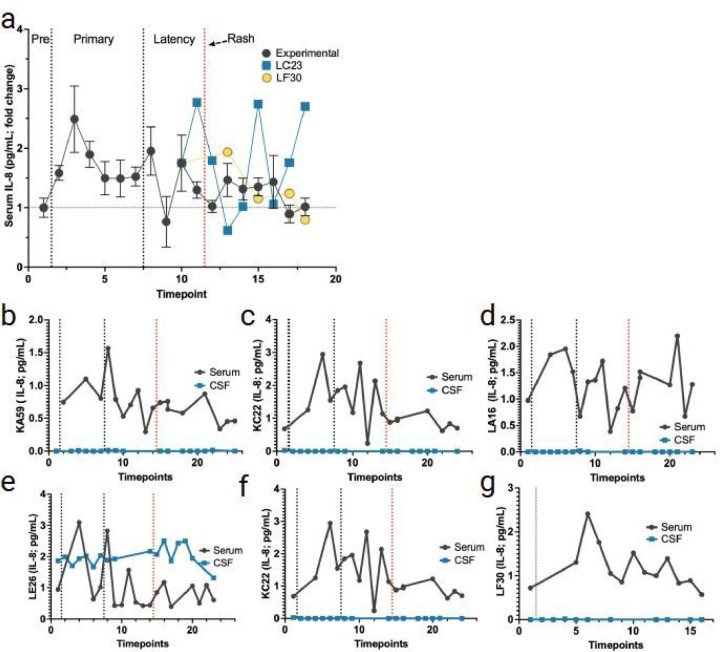
Changes in IL-8 in serum and CSF following SVV primary SVV infection, latency, and reactivation. **a** average fold change in serum IL-8 (average for all RMs) from pre-SVV inoculation (Pre) for primary infection and latency. All animals were treated the same during primary infection; only at transportation (timepoint 10) do LC23 and LF30 differ experimentally. **b-e** averaged fold change in serum (black) and CSF (blue) IL-8 for the four experimental RMs (KA59, KC22, LA16, LE26) following immunosuppression and reactivation. **e**levels of CSF IL-10 were only detected in one RM (LE26). **f, g** averaged fold change in serum and CSF IL-8 shown for LC23 (non-immunosuppressed) and LF30 (latently infected). Individual serum/CSF RM IDs are shown on the Y-axis. Individual serum, or CSF IL-8 levels not detected, are indicated as 0 in individual graphs.

Serum IL-8 was increased in all RMs during primary infection and remained high until latency (Fig. 7a). All animals, including the two control monkeys, had elevated IL-8 levels in serum during primary infection (Fig. 7a–g). One experimental animal (LA16) and the transportation control (LC32) showed increased serum IL-8 following zoster (Fig. 7d, f). The non-immunosuppressed monkey (LC23) also had elevated IL-8 just prior to reactivation (Fig. 7f). Only one experimental animal (LE26) showed any detectable IL-8 in the CSF, and these levels stayed consistent throughout primary infection, latency, and reactivation (Fig. 7e).

### Changes in Aβ40 and Aβ42 peptides in the serum and CSF during primary infection, latency, and reactivation.

**Figure 8:**
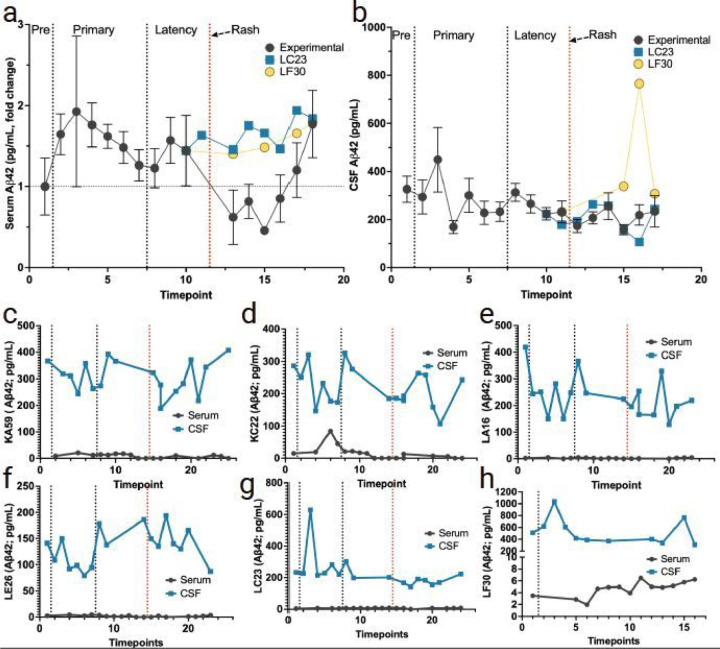
Changes in levels of Aβ42 peptide in serum and CSF following primary SVV infection, latency, and reactivation. **a** fold change in serum levels of Aβ42 peptide (average for all RMs) from pre-SVV inoculation (Pre) for primary infection and latency. **b**Means in CSF levels of Aβ42 peptides (average for all monkeys) from pre-SVV inoculation, primary infection, and latency. All animals were treated the same during primary infection; only at transportation (timepoint 10) do LC23 and LF30 differ experimentally. **c-f** averaged fold change in the serum (black), and CSF (blue) levels of Aβ42 peptide can be seen for the four experimental RMs (KA59, KC22, LA16, LE26) following immunosuppression and reactivation. **g, h** serum and CSF Levels of Aβ40 peptide are shown separately in control monkeys LC23 (non-immunosuppressed) and LF30 (latently infected). Individual serum/CSF monkey IDs are shown on the Y-axis. Individual serum or CSF Aβ42 levels not detected are indicated as 0 in individual graphs.

**Figure 9:**
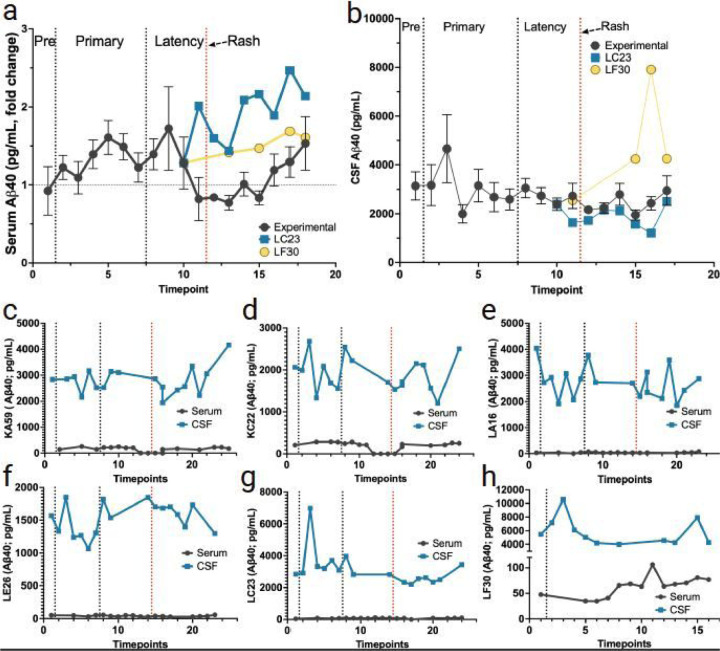
Changes in levels of Aβ40 peptide in serum and CSF following primary SVV infection, latency, and reactivation. **a** fold change in serum levels of Aβ40 peptides (average for all RMs) from pre-SVV inoculation (Pre) for primary infection and latency. **b** Mean CSF levels of Aβ40 peptides (for all RMs) from pre-SVV inoculation, primary infection, and latency. All RMs were treated the same during primary infection; only at transportation (timepoint 10) do LC23 and LF30 differ experimentally. **c-f** averaged fold change in the serum (black), and CSF (blue) levels of Aβ40 peptide can be seen for the four experimental RMs (KA59, KC22, LA16, LE26) following immunosuppression and reactivation. **g, h** Serum and CSF Levels of Aβ40 peptide are shown separately in control RMs LC23 (non-immunosuppressed) and LF30 (latently infected). Individual RM Individual monkey IDs are shown on the Y-axis. Individual serum or CSF Aβ40 levels not detected are indicated as 0 in individual graphs.

A role for Aβ42 and Aβ40 in the host’s anti-microbial response has been previously proposed (Schwartz and Boles, 2013; Soscia et al., 2010). Therefore, we investigated the levels of Aβ42 and Aβ40 in both the periphery and CNS during primary SVV infection, latency, and reactivation (Fig. 8, 9). Serum Aβ42 and Aβ40 levels were slightly elevated during primary SVV infection (Fig. 8a, 9a). However, during reactivation, we observed a decrease in both Aβ42 and Aβ40 levels in the serum. A similar trend followed with slightly elevated Aβ42 and Aβ40 during primary infection and a slight decrease during reactivation (Fig. 8c–h and Fig. 9c–h). The non-immunosuppressed monkey (LC23) that developed zoster showed a similar trend as experimental animals, with a decrease in serum Aβ42 and Aβ40 following reactivation. The latently infected control monkey (LF30) had the opposite effect, with a shared transient increase of CSF Aβ42 and Aβ40 during primary infection and latency.

## Discussion

This study aimed to describe the concurrent peripheral serum and CSF cytokine and Aβ42/Aβ40 responses during primary infection, latency, and reactivation of SVV-infected RMs compared to pre-inoculation levels to better understand the interplay between the periphery and CNS during VZV pathogenesis. Our data revealed a robust pro-inflammatory response in the serum and CSF during primary SVV infection and reactivation. Our results confirm and extend previous reports of increased cytokines within plasma of RMs during primary SVV infection and reactivation (Haberthur et al. 2013; Meyer et al. 2013; Traina-Dorge et al. 2014). We found high levels of cytokines, including IL-6, IL-8, IL-10, and IL-2, during primary infection and, while more variable, also found elevated levels during reactivation. Interestingly, we also found elevated cytokines within the CSF during primary infection and reactivation. However, these levels did not always correspond to elevated levels in the periphery. We also found elevated levels of both Aβ40 and Aβ following primary infection in the plasma, while there was an overall decrease following zoster. Levels of both peptides in the CSF were slightly elevated during primary infection and had a small decrease during zoster.

The results showed that serum IL-6 levels spiked during primary infection in all animals, while CSF IL-6 levels exhibited only in three out of six animals were elevated (Fig. 3). Animals KA59, LA16, and treatment control RM LC23 showed spikes in CSF IL-6 that aligned with serum IL-6 levels during primary infection. Animals KC22 and LE26 had fluctuations in serum IL-6 levels but minimal change in CSF IL-6. Interestingly, the control RM LC23 had a large spike in CSF IL-6 during reactivation without substantial changes in serum IL-6 levels. IL-6 has a variety of actions, including both pro- and anti-inflammatory innate immune responses (Scheller et al. 2011; Schneiders et al. 2015). Increases in IL-6 transcription during VZV-skin infection is well documented and may be considered a cellular stress response in the periphery (Jarosinski et al. 2018). CSF of patients with VZV vasculopathy had elevated IL-6 and IL-8, which may increase the risk of vascular disease (Jones et al. 2016). Serum IL-8 levels increased during primary infection and remained high until latency in all animals (Fig 7). One experimental animal and the transportation control LF30 showed a second spike in serum IL-8 following zoster. Non-immunosuppressed RM, LC23, also had elevated IL-8 levels prior to reactivation. Only one experimental animal showed detectable IL-8 in the CSF throughout the infection. Furthermore, studies have shown that both IL-6 and IL-8 were elevated in VZV-infected vascular cells and are known to promote neutrophil and macrophage activation in VZV-infected arteries (Jones et al. 2017). Taken together, the increase in IL-6 and IL-8 may suggest that those specific RMs may have an increased risk of CNS disease, including stroke or vasculopathy that would have otherwise gone undetected within the periphery.

Both IL-2 and IL-10 levels showed (how many) elevated spikes in serum during primary infection (Fig. 4, 5). CSF IL-2 levels remained largely unchanged in these animals, except for LA16, which exhibited a spike in CSF IL-2 before the onset of zoster (Fig. 4e). Non-immunosuppressed monkey LC23 also had a spike in CSF IL-2 during primary infection (Fig. 4g). Control animals LC23 and LF30 displayed spikes in CSF IL-10 during primary infection (Fig. 5). CSF levels of IL-2 in three experimental animals did not change, even with elevated peripheral IL-2. IL-2 presence is indicative of a T-cell activation/inflammatory process (Lan et al. 2008; Valbon et al. 2016). Elevated soluble IL-2 receptors have been found in patients with multiple sclerosis, stroke, and systemic lupus erythematous (El-Shafey et al. 2008; Maier et al. 2009; Peerlings et al. 2021; Zhao et al. 2020).

IL-1β plays a critical host-defense mechanism early in infection (Lopez-Castejon and Brough, 2011; Nour et al. 2011). However, serum IL-1β levels only slightly increased during primary infection and reactivation in all experimental animals (Fig. 6). CSF IL-1β increased during latency before zoster onset in all experimental animals. Notably, animal KA59 had a large spike in CSF right before the onset of zoster rash and proceeded the spike in serum IL-1β (Fig 6c). Control animals LC23 and LF30 exhibited the largest spike in serum IL-1β during primary infection, with LC23 showing an additional spike following zoster (Fig 6g, h) The variability in these results is surprising given IL-1β is important for initial host defense. However, clinical data suggest higher CSF levels of IL-1β in patients who develop post-herpetic neuralgia, suggesting a role for elevated CNS inflammatory markers and the development of chronic pain disorders associated with infection (Zhao et al. 2017).

The study also investigated the levels of Aβ42 and Aβ40 in the serum and CSF. Serum Aβ42 and Aβ40 levels were slightly elevated during primary infection and decreased during reactivation. A similar trend was observed in CSF Aβ42 and Aβ40 levels, except for the latently infected control monkey LF30, which showed a transient increase during both primary infection and latency. Measurements of CSF and plasma Aβ42 and Aβ40 peptide solubility are suggestive of clearance from the brain, and individuals with Alzheimer’s disease routinely have less Aβ42 and Aβ40 in both the CSF and plasma (Dorey et al. 2015; Mehta et al. 2000). Indeed about 30–50% of Aβ peptides found in the blood are transported from the CNS via transport through the blood-brain barrier (Ovod et al. 2017; Roberts et al. 2014; Tanzi et al. 2004). A recent study found that plasma Aβ42 and Aβ40 ratios decreased by 15% in amyloid-positive patients compared to amyloid-negative patients (Klafki et al. 2022). It is of note that we found the largest change in Aβ42 and Aβ40 within the serum levels, with relatively less change within the CSF. These findings may be indicative of total clearance of Aβ42 and Aβ40 from the CNS into the bloodstream. Taken together, these findings suggest a potential attenuation of Aβ42 and Aβ40 clearance during zoster.

Overall, the results suggest differences in cytokine and Aβ42/Aβ40 levels between the periphery and CNS during SVV infection in RMs. The findings provide insights into the immune response and potential involvement of cytokines and amyloidogenic peptides in the pathogenesis of SVV infection within the CNS that may otherwise be missed in peripheral assessments of VZV-associated diseases.

## Supplementary Material

Supplement 1

## References

[1] Dorey A, Perret-Liaudet A, Tholance Y, Fourier A, Quadrio I (2015) Cerebrospinal Fluid Abeta40 Improves the Interpretation of Abeta42 Concentration for Diagnosing Alzheimer’s Disease. Front Neurol 6:247 26640457 10.3389/fneur.2015.00247 PMC4661235

[2] El-Shafey EM, El-Nagar GF, El-Bendary AS, Sabry AA, Selim AG (2008) Serum soluble interleukin-2 receptor alpha in systemic lupus erythematosus. Iran J Kidney Dis 2:80–85 19377213

[3] Gershon AA, Breuer J, Cohen JI, Cohrs RJ, Gershon MD, Gilden D, Grose C, Hambleton S, Kennedy PGE, Oxman MN, Seward JF, Yamanishi K (2015) Varicella zoster virus infection. Nat Reviews Disease Primers 1:15016 10.1038/nrdp.2015.16 PMC5381807 27188665

[4] Gilden D, Cohrs RJ, Mahalingam R, Nagel MA (2010) Neurological disease produced by varicella zoster virus reactivation without rash. Curr Top Microbiol Immunol 342:243–253 20186614 10.1007/82_2009_3 PMC3076592

[5] Haberthur K, Kraft A, Arnold N, Park B, Meyer C, Asquith M, Dewane J, Messaoudi I (2013) Genome-Wide Analysis of T Cell Responses during Acute and Latent Simian Varicella Virus Infections in Rhesus Macaques. J Virol 87:11751–11761 23986583 10.1128/JVI.01809-13 PMC3807353

[6] Jarosinski KW, Carpenter JE, Buckingham EM, Jackson W, Knudtson K, Moffat JF, Kita H, Grose C (2018) Cellular Stress Response to Varicella-Zoster Virus Infection of Human Skin Includes Highly Elevated Interleukin-6 Expression. Open Forum Infect Dis 5:ofy118 30014002 10.1093/ofid/ofy118 PMC6007511

[7] Jones D, Alvarez E, Selva S, Gilden D, Nagel MA (2016) Proinflammatory cytokines and matrix metalloproteinases in CSF of patients with VZV vasculopathy. Neurol - Neuroimmunol Neuroinflammation 3:e246 10.1212/NXI.0000000000000246 PMC4907802 27340684

[8] Jones D, Neff CP, Palmer BE, Stenmark K, Nagel MA (2017) Varicella zoster virus–infected cerebrovascular cells produce a proinflammatory environment. Neurol - Neuroimmunol Neuroinflammation 4:e382 10.1212/NXI.0000000000000382 PMC5682918 29159203

[9] Klafki H-W, Morgado B, Wirths O, Jahn O, Bauer C, Esselmann H, Schuchhardt J, Wiltfang J (2022) Is plasma amyloid-β 1–42/1–40 a better biomarker for Alzheimer’s disease than AβX–42/X–40? Fluids and Barriers of the CNS 19. 10.1186/s12987-022-00390-4 PMC9719149 36461122

[10] Lan RY, Selmi C, Gershwin ME (2008) The regulatory, inflammatory, and T cell programming roles of interleukin-2 (IL-2). J Autoimmun 31:7–12 18442895 10.1016/j.jaut.2008.03.002

[11] Lopez-Castejon G, Brough D (2011) Understanding the mechanism of IL-1beta secretion. Cytokine Growth Factor Rev 22:189–195 22019906 10.1016/j.cytogfr.2011.10.001 PMC3714593

[12] Mahalingam R, Feia B, Coleman C, Anupindi K, Saravanan P, Luthens A, Bustillos A, Das A, de Haro E, Doyle-Meyers L, Looper J, Bubak AN, Niemeyer CS, Palmer B, Nagel MA, Traina-Dorge V (2022) Simian Varicella Virus Pathogenesis in Skin during Varicella and Zoster. Viruses 14 10.3390/v14061167 PMC9227806 35746639

[13] Mahalingam R, Wellish M, Soike K, White T, Kleinschmidt-DeMasters BK, Gilden DH (2001) Simian varicella virus infects ganglia before rash in experimentally infected monkeys. Virology 279:339–342 11145914 10.1006/viro.2000.0700

[14] Mahalingam R, Wellish M, Wolf W, Dueland AN, Cohrs R, Vafai A, Gilden D (1990) Latent varicella-zoster viral DNA in human trigeminal and thoracic ganglia. N Engl J Med 323:627–631 2166914 10.1056/NEJM199009063231002

[15] Maier LM, Anderson DE, Severson CA, Baecher-Allan C, Healy B, Liu DV, Wittrup KD, De Jager PL, Hafler DA (2009) Soluble IL-2RA Levels in Multiple Sclerosis Subjects and the Effect of Soluble IL-2RA on Immune Responses. J Immunol 182:1541–1547 19155502 10.4049/jimmunol.182.3.1541 PMC3992946

[16] Mehta PD, Pirttila T, Mehta SP, Sersen EA, Aisen PS, Wisniewski HM (2000) Plasma and cerebrospinal fluid levels of amyloid beta proteins 1–40 and 1–42 in Alzheimer disease. Arch Neurol 57:100–105 10634455 10.1001/archneur.57.1.100

[17] Messaoudi I, Barron A, Wellish M, Engelmann F, Legasse A, Planer S, Gilden D, Nikolich-Zugich J, Mahalingam R (2009) Simian Varicella Virus Infection of Rhesus Macaques Recapitulates Essential Features of Varicella Zoster Virus Infection in Humans. PLoS Pathog 5:e1000657 19911054 10.1371/journal.ppat.1000657 PMC2770849

[18] Meyer C, Kerns A, Haberthur K, Dewane J, Walker J, Gray W, Messaoudi I (2013) Attenuation of the adaptive immune response in rhesus macaques infected with simian varicella virus lacking open reading frame 61. J Virol 87:2151–2163 23221560 10.1128/JVI.02369-12 PMC3571457

[19] Nagel MA, Niemeyer CS, Bubak AN (2020) Central nervous system infections produced by varicella zoster virus. Curr Opin Infect Dis 33:273–278 32332223 10.1097/QCO.0000000000000647 PMC13183292

[20] Nour AM, Reichelt M, Ku C-C, Ho M-Y, Heineman TC, Arvin AM (2011) Varicella-Zoster Virus Infection Triggers Formation of an Interleukin-1β (IL-1β)-processing Inflammasome Complex. J Biol Chem 286:17921–17933 21385879 10.1074/jbc.M110.210575 PMC3093867

[21] Ovod V, Ramsey KN, Mawuenyega KG, Bollinger JG, Hicks T, Schneider T, Sullivan M, Paumier K, Holtzman DM, Morris JC, Benzinger T, Fagan AM, Patterson BW, Bateman RJ(2017). Amyloid β concentrations and stable isotope labeling kinetics of human plasma specific to central nervous system amyloidosis. Alzheimer’s & Dementia 13: 841–849 10.1016/j.jalz.2017.06.2266 PMC5567785 28734653

[22] Peerlings D, Mimpen M, Damoiseaux J (2021) The IL-2 - IL-2 receptor pathway: Key to understanding multiple sclerosis. J Transl Autoimmun 4:100123 35005590 10.1016/j.jtauto.2021.100123 PMC8716671

[23] Richter ER, Dias JK, Gilbert JE 2nd, Atherton SS (2009) Distribution of herpes simplex virus type 1 and varicella zoster virus in ganglia of the human head and neck. J Infect Dis 200:1901–1906 19919304 10.1086/648474 PMC2782560

[24] Roberts KF, Elbert DL, Kasten TP, Patterson BW, Sigurdson WC, Connors RE, Ovod V, Munsell LY, Mawuenyega KG, Miller-Thomas MM, Moran CJ, Cross DT, Derdeyn CP, Bateman RJ (2014) Amyloid-β efflux from the central nervous system into the plasma. Ann Neurol 76:837–844 25205593 10.1002/ana.24270 PMC4355962

[25] Scheller J, Chalaris A, Schmidt-Arras D, Rose-John S (2011) The pro- and anti-inflammatory properties of the cytokine interleukin-6. Biochim Biophys Acta 1813:878–888 21296109 10.1016/j.bbamcr.2011.01.034

[26] Schneiders J, Fuchs F, Damm J, Herden C, Gerstberger R, Soares DM, Roth J, Rummel C (2015) The transcription factor nuclear factor interleukin 6 mediates pro- and anti-inflammatory responses during LPS-induced systemic inflammation in mice. Brain Behav Immun 48:147–164 25813145 10.1016/j.bbi.2015.03.008

[27] Schwartz K, Boles BR (2013) Microbial amyloids - functions and interactions within the host. Curr Opin Microbiol 16:93–99 23313395 10.1016/j.mib.2012.12.001 PMC3622111

[28] Soscia SJ, Kirby JE, Washicosky KJ, Tucker SM, Ingelsson M, Hyman B, Burton MA, Goldstein LE, Duong S, Tanzi RE, Moir RD (2010) The Alzheimer’s disease-associated amyloid beta-protein is an antimicrobial peptide. PLoS ONE 5:e9505 20209079 10.1371/journal.pone.0009505 PMC2831066

[29] Tanzi RE, Moir RD, Wagner SL (2004) Clearance of Alzheimer’s Abeta peptide: the many roads to perdition. Neuron 43:605–608 15339642 10.1016/j.neuron.2004.08.024

[30] Traina-Dorge V, Sanford R, James S, Doyle-Meyers LA, De Haro E, Wellish M, Gilden D, Mahalingam R (2014) Robust pro-inflammatory and lesser anti-inflammatory immune responses during primary simian varicella virus infection and reactivation in rhesus macaques. J Neurovirol 20:526–530 25139181 10.1007/s13365-014-0274-2 PMC4394654

[31] Valbon SF, Condotta SA, Richer MJ (2016) Regulation of effector and memory CD8(+) T cell function by inflammatory cytokines. Cytokine 82:16–23 26688544 10.1016/j.cyto.2015.11.013

[32] Zerboni L, Sen N, Oliver SL, Arvin AM (2014) Molecular mechanisms of varicella zoster virus pathogenesis. Nat Rev Microbiol 12:197–210 24509782 10.1038/nrmicro3215 PMC4066823

[33] Zhao H, Li F, Huang Y, Zhang S, Li L, Yang Z, Wang R, Tao Z, Han Z, Fan J, Zheng Y, Ma Q, Luo Y (2020) Prognostic significance of plasma IL-2 and sIL-2Rα in patients with first-ever ischaemic stroke. J Neuroinflamm 17 10.1186/s12974-020-01920-3 PMC7427726 32795376

[34] Zhao WX, Wang Y, Fang QQ, Wu JP, Gao XY, Liu H, Cao L, An JX (2017) Changes in neurotrophic and inflammatory factors in the cerebrospinal fluid of patients with postherpetic neuralgia. Neurosci Lett 637:108–113 27888042 10.1016/j.neulet.2016.11.041

