# Supplementary material for: Simian Varicella Virus Infection and Reactivation in Rhesus Macaques Trigger Cytokine and Aβ40/42 Alterations in Serum and Cerebrospinal Fluid
Source: Res Sq. 2023 Oct 16:rs.3.rs-3367215. Preprint. [Version 1] doi: 10.21203/rs.3.rs-3367215/v1 (PMC10602180; doi:10.21203/rs.3.rs-3367215/v1)
Supplement: Supplement 1 [file NIHPPrs3367215v1-supplement-1.pdf]

Supplementary Files

This is a list of supplementary files associated with this preprint. Click to download.

- SupTable1KA59.pdf
- SupTable2KC22.pdf
- SupTable3LA16.pdf
- SupTable4LE26.pdf
- SupTable5KC22.pdf
- SupTable6LF30.pdf
